# Suboptimal movement behaviours among children under two years old in early childhood education institutions in urban China: A cross-sectional study

**DOI:** 10.7189/jogh.13.04110

**Published:** 2023-09-29

**Authors:** Xiaotong Wang, Yiwen Huang, Na Meng, Jian Zhang, Qiong Wu, Yanfeng Zhang

**Affiliations:** 1 Department of Integrated Early Childhood Development, Capital Institute of Pediatrics, Beijing, China; 2 Child Healthcare Center, Children’s Hospital, Capital Institute of Pediatrics, Beijing, China

## Abstract

**Background:**

Healthy movement behaviours in early life promote better physical health and facilitate psychological development. Early childhood education (ECE) institutions provide opportunities for caregivers to improve children’s movement behaviour practices, but little is known about the current status of movement behaviours among infants and young children in urban China in this context. We aimed to investigate the movement behaviour status of children under two years of age via ECE institutions and compare movement behaviour practices and knowledge between children attending ECE classes and those not attending.

**Methods:**

We conducted the cross-sectional survey from 1 March to 30 April 2023 in Gymboree Play&Music, an ECE institution covering 31 provinces in China. We developed our survey instrument based on World Health Organization (WHO) guidelines to measure children’s movement behaviour practices and caregivers’ movement behaviour knowledge. We carried out the online questionnaire survey through Sojump, the largest professional online survey platform in China. We sent a quick response (QR) code to Gymboree Play&Music centres; the QR code was linked to a questionnaire intended for caregiver members and non-members coming for experience classes and activities.

**Results:**

We surveyed 3355 primary caregivers, encompassing 504, 536, and 2315 children aged 0-5 months, 6-11 months, and 12-23 months, respectively. Overall, less than half of the children met recommendations in physical activity time (PAT) (19.2%), physical restraint (PR) (45.8%), and screen time (ST) (46.4%) during the last 24 hours. PAT, outdoor time (OT), and sleep duration (SD) in children aged 0-5 months were significantly lower than in the other two age groups, while ST was significantly higher than in the other two age groups during the last 24 hours (all *P* < 0.001). For children attending ECE classes, PAT, OT, and SD were significantly higher than those not attending ECE during the last 24 hours and the last two weeks (all *P* < 0.05). All indicators in the long-nursing care time (NCT) group were significantly higher than those in the short-NCT group, while there was no statistical difference in SD between the two groups (66.6% vs 65.6%; *P* = 0.558).

**Conclusions:**

The status of movement behaviours for children under two years old in urban China is not optimistic, especially for PAT, PR, and ST. Additionally, attending early childhood education classes and primary caregivers’ daily nurturing care time are important for infants and young children to adhere to movement guidelines. Consequently, more ECE institution-specific practical strategies and educational materials are needed to promote compliance with movement behaviour guidelines.

Healthy movement behaviours, including sufficient physical activity, reduced sedentary behaviours, and optimal sleep duration, have important health implications during childhood and adulthood through trajectory effects [1-4]. Evidence indicates that the interaction, of all three healthy movement behaviours not only reduces the prevalence of obesity, myopia, and the risk of cardiovascular diseases but also has continuous effects on psychosocial outcomes and cognitive development [5-8]. During the first two years of life, infants and young children begin to acquire and rapidly improve motor control and ability, while movement habits developed during this period tend to carry over into later childhood, adolescence, and adulthood [9]. Therefore, it is essential healthy movement behaviours in children under two years old are promoted.

The World Health Organization (WHO) and many other countries have recently issued movement behaviour guidelines or consensus statements for the early years of life, highlighting the importance of healthy movement behaviours to maximize the health benefits [3,5,10-16]. Based on the WHO guidelines, China also released a physical activity guideline in 2021, which include recommendations for children under two years old [17]. Documenting data on compliance with movement behaviour guidelines is important for effective public health interventions and surveillance [18]. In China, surveys on movement behaviours are mainly conducted among preschool children and teenagers [19-21], and there is limited data on movement behaviour practices for infants and young children. Data from our previous study indicated that the current status of healthy movement behaviours among children aged six to 20 months was suboptimal [22]. However, the survey was only carried out in a single county in Qinghai Province, and there is no representative national data on movement behaviours for children under two years old in urban and rural China, highlighting a need for more comprehensive data collection.

Primary caregivers are essential for establishing healthy movement behaviours for infants and young children [23]. Evidence shows that parental knowledge and attitude appear to be crucial for offspring’s physical activity practices, while parents’ screen time and sleep duration are also related to offspring’s screen time and sleep duration [24,25]. With the development of the social economy and continuous improvement of people's lives, an increasing number of Chinese parents have started to add importance to their children's early childhood education (ECE), especially in urban China. Approximately 40% of children under three years old in Shanghai attended ECE institutions [26], which generally provide children with rich learning experiences to maximise motor, cognitive, and other development [27]. These institutions thus offer an opportunity to improve caregivers’ movement behaviour knowledge and promote children's movement behaviour practices. However, no studies investigated the current status of children's movement behaviour through ECE institutions in China. Therefore, we aimed to investigate the movement behaviour status of children under two years old in urban China via ECE institutions and to explore factors which may affect children’s movement behaviour status.

## METHODS

### Study design and participants

We conducted a cross-sectional study across 31 provinces in China from 1 March to 30 April 2023, in collaboration with Gymboree Play&Music, an ECE institution with over 500 centres in nearly 200 cities in urban China. We used WeChat self-administered questionnaires to collect data on movement behaviour practices and knowledge from children and their primary caregivers.

We included primary caregivers who were Gymboree Play&Music members with their children attending ECE classes and non-Gymboree Play&Music members coming only for experience classes and activities. We excluded caregivers if they had no WeChat app on their smartphones, had children older than 23 months, or if they refused to participate in the survey. We categorised children into three age groups: 0-5 months, 6-11 months and 12-23 months. We defined children as having attended ECE classes during the last month if they attend ECE classes at least once per week during the last month. Moreover, we categorised primary caregivers’ daily nurturing care time during the last month into two groups (<4 hours and ≥4 hours) [28].

### Sample size and sampling

We calculated our sample size based on our previous study [22], expecting the proportion of children who meet recommended physical activity time during the last 24 hours at 23.0% and a desired level of absolute precision of 2.5%. With a 5% significance and 80% power, we estimated that we required 2273 participants; considering a 30% response loss, we proposed recruiting 3247 primary caregivers of children under two years old. We used convenience sampling method to recruit primary caregivers with the assistance of ECE teachers of Gymboree Play&Music.

### Survey instrument

We developed our survey instrument based on the WHO guidelines on physical activity, sedentary behaviour, and sleep for children under five years of age to measure children’s movement behaviour practices and caregivers’ movement behaviour knowledge, which consisted of physical activity time (PAT), outdoor time (OT), physical restraint (PR), sleep duration (SD), and screen time (ST) of children during the last 24 hours and the last two weeks. We have used the survey instrument in our previous study [22]. Furthermore, we collected information on how many times children attended ECE classes per week during the last month, the duration for each class, and caregivers’ nurturing care time spent on children every day during the last week. We set up the questionnaire on the Sojump platform, the largest professional online survey platform in China, and then obtained a quick response (QR) code linked to the questionnaire.

### Data collection

**Figure 1:**
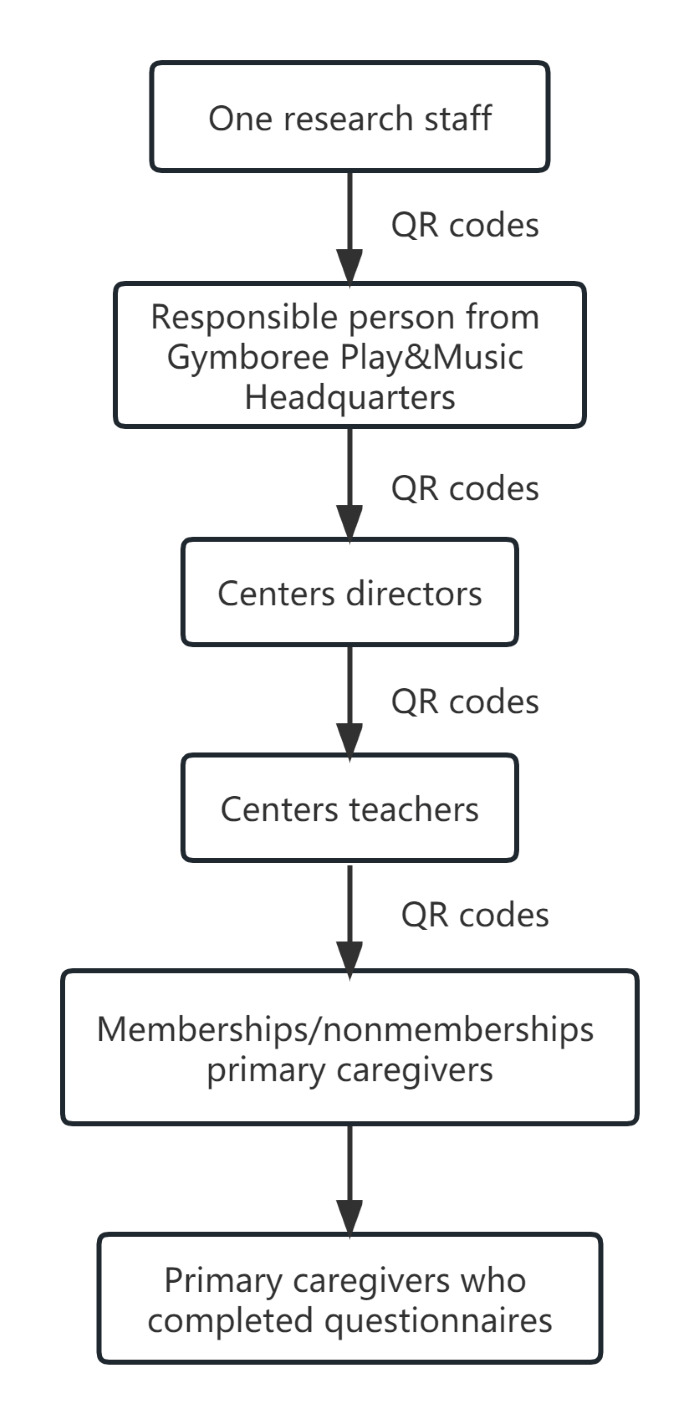
Study process.

We collected data with the help of Gymboree Play&Music. One researcher (WXT) first sent the QR code of the WeChat questionnaire to the responsible person from Gymboree Play&Music headquarters, followed by centre directors and then centre teachers (**Figure 1**). Teachers in each centre invited primary caregivers to participate in the survey and sent a QR code to them after obtaining their oral consent. Primary caregivers scanned the QR code using their WeChat app and filled in the questionnaire online.

### Outcomes

The primary outcomes were the proportions of children who met the WHO recommendations of PAT, OT, PR, SD, and ST during the last 24 hours and the last two weeks by three age groups (**Box 1**). The secondary outcomes were the proportions of primary caregivers who knew PAT, OT, PR, SD, and ST recommendations by three age group, the differences in proportions of children’s movement behaviour practices and caregivers’ movement behaviour knowledge between children who attended ECE classes (attending group) and those who did not attend ECE classes (non-attending group) during the last month, and the differences in proportions of children’s movement behaviour practices and caregivers’ movement behaviour knowledge between children whose primary caregivers’ daily nurturing care time ≥4 hours (long NCT group) and those whose primary caregivers’ daily nurturing care time <4 hours (short NCT group) during the last month.

Box 1 **Movement behaviour assessment**

The primary outcomes for movement behaviour assessment were indicators based on the WHO recommendations.

1. **Physical activity time (PAT):** percentage of children aged 0-23 months whose physical activity time met the recommendation during the last 24 hours and two weeks.
  1. For children aged 0-11 months: spend at least 120 minutes per day in various types of physical activities, including tummy time, crawling, and arm and leg movements.
  2. For children aged 12-23 months: spend at least 180 minutes per day in various types of physical activities, including crawling, standing and walking.
2. **Outdoor time (OT):** percentage of children aged 0-23 months whose outdoor time met the recommendation during the last 24 hours and two weeks.
  1. For children aged 0-11months: outdoor activities at least once daily.
  2. For children aged 12-23 months: spend at least 60 minutes per day for outdoor activities.
3. **Physical restraint (PR):** percentage of children aged 0-23 months who met the recommendation on physical restraint during the last 24 hours and two weeks.
  1. For children aged 0-23 months: not be restrained for more than one hour at a time (e.g. prams/strollers, highchairs or strapped on a caregiver’s back) or sit for extended periods.
4. **Sleep duration (SD):** percentage of children aged 0-23 months whose sleep duration met the recommendations during the last 24 hours and two weeks.
  1. For children aged 0-3 months: have 14-17 hours of good quality sleep, including naps; for children aged 4-11 months: have 12-16 hours of good quality sleep, including naps.
  2. For children aged 12-23 months: have 11-14 hours of good quality sleep, including naps.
5. **Screen time (ST):** percentage of children aged 0-23 months whose screen time met the recommendations during the last 24 hours and two weeks.
  1. For children aged 0-23 months: have no screen time (such as watching TV/videos or playing computer games).

### Data management and statistical analysis

Once caregivers completed and submitted the questionnaire online, their answers were uploaded to the database of the Sojump platform. One researcher (WXT) oversaw the Sojump account and secured the database. The database was downloaded locally and converted into Microsoft Excel sheets for data analysis.

We used R, version 4.1.2. (R Core Team, Auckland, New Zealand) for statistical analysis [29]. We presented demographic and movement behaviour outcomes in tables and graphs using descriptive statistics (numbers and proportions) by three age groups. We used a χ^2^ test to determine the significance of categorical variables among age groups, with *P* < 0.05 denoting statistical significance.

### Ethical approval

The Ethical Committee of the Capital Institute of Pediatrics in Beijing (SHERLL2022041) approved this study. Each WeChat questionnaire contained an electronic informed consent which the participating caregivers read and clicked “Agree to participate” before they answered the questions.

## RESULTS

### Location and sample size

**Figure 2:**
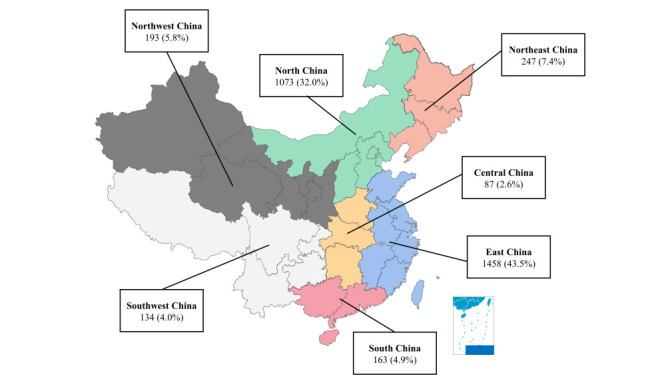
Location map and sample size.

We surveyed 3355 primary caregivers from seven geographical areas, primarily East China (43.5%) and North China (32.0%), while less than one-fourth came from the other five geographical areas of China (**Figure 2**).

### Socio-demographic characteristics

We included 504 children aged 0-5 months, 536 children aged 6-11 months, and 2315 children aged 12-23 months in the analysis; nearly three-fourths attended ECE classes during the last month, with children aged 0-5 months attending much less than the other two groups (*P* < 0.001). The primary caregivers were mostly mothers (75.4%), followed by fathers (17.9%) and grandparents (4.2%). Most primary caregivers (87.0%) were college-educated or above, and 62.1% of them spent more than four hours on nurturing care for their children every day during the last month (**Table 1**).

**Table 1:** Characteristics of surveyed children and their primary caregivers*

	0-5 mo (n = 504)	6-11 mo (n = 536)	12-23 mo (n = 2315)	Total (n = 3355)	*P*-value†
**Children**					
Sex					
*Male*	248 (49.2)	266 (49.6)	1104 (47.7)	1618 (48.2)	0.643
*Female*	256 (50.8)	270 (50.4)	1211 (52.3)	1737 (51.8)	
Ethnicity					
*Han*	464 (92.1)	494 (92.2)	2185 (94.4)	3143 (93.7)	0.044
*Ethnic minorities*	40 (7.9)	42 (7.8)	130 (5.6)	212 (6.3)	
Attended ECE classes during the last month	302 (59.9)	417 (77.8)	1771 (76.5)	2490 (74.2)	<0.001
**Primary caregivers**					
Relationship					
*Mother*	354 (70.2)	394 (73.5)	1781 (76.9)	2529 (75.4)	0.002
*Father*	105 (20.8)	104 (19.4)	391 (16.9)	600 (17.9)	
*Grandparents*	30 (6.0)	31 (5.8)	80 (3.5)	141 (4.2)	
*Others*	15 (3.0)	7 (1.3)	63 (2.7)	85 (2.5)	
Education					
*Senior high school or below*	67 (13.3)	88 (16.4)	280 (12.1)	435 (13.0)	0.027
*College or above*	437 (86.7)	448 (83.6)	2035 (87.9)	2920 (87.0)	
Primary caregivers’ daily nurturing care time during the last month					
*≥4 h/d*	324 (64.3)	318 (59.3)	1442 (62.3)	2084 (62.1)	0.246
*<4 h/d*	180 (35.7)	218 (40.7)	873 (37.7)	1271 (37.9)	

*Values presented as n (%) unless specified differently.

†χ^2^ test for comparing differences in proportion of three age groups.

### Practices and knowledge of movement behaviours

Movement behaviour practices were generally suboptimal: less than 50% of total children met the WHO recommendations on PAT (19.2%), PR (45.8%) and ST (46.4%), during the last 24 hours. PAT, OT, and SD in children aged 0-5 months were significantly lower than in the other two age groups (*P* < 0.001), while ST was significantly higher than in the other two age groups during the last 24 hours (*P* < 0.001). Movement behaviour practice indicators during the last two weeks showed similar patterns. Caregivers’ knowledge of movement behaviours was also suboptimal: only 15% of all caregivers knew the PAT recommendations, and around 50%-70% of them knew the recommendations of the other four indicators (**Table 2**).

**Table 2:** Status of movement behaviours of children under two years old in urban China*

	0-5 mo (n = 504)	6-11 mo (n = 536)	12-23 mo (n = 2315)	Total (n = 3355)†
**Movement behaviour practices during the last 24 h**				
Physical activity time	55 (10.9)	124 (23.1)	466 (20.1)	645 (19.2)
Outdoor time	386 (76.6)	485 (90.5)	2005 (86.6)	2876 (85.7)
Physical restraint	213 (42.3)	184 (34.3)	1141 (49.3)	1538 (45.8)
Sleep duration	292 (57.9)	401 (74.8)	1522 (65.7)	2215 (66.0)
Screen time	352 (69.8)	319 (59.5)	886 (38.3)	1557 (46.4)
**Movement behaviour practices during the last two weeks**				
Physical activity time	78 (15.5)	132 (24.6)	516 (22.3)	726 (21.6)
Outdoor time	418 (82.9)	507 (94.6)	2070 (89.4)	2995 (89.3)
Physical restraint	197 (39.1)	145 (27.1)	905 (39.1)	1247 (37.2)
Sleep duration	280 (55.6)	397 (74.1)	1502 (64.9)	2179 (64.9)
Screen time	356 (70.6)	323 (60.3)	822 (35.5)	1501 (44.7)
**Movement behaviour knowledge (caregivers know recommendations)**				
Physical activity time	39 (7.7)	87 (16.2)	377 (16.3)	503 (15.0)
Outdoor time	413 (81.9)	487 (90.9)	1118 (48.3)	2018 (60.1)
Physical restraint	329 (65.3)	345 (64.4)	1746 (75.4)	2420 (72.1)
Sleep duration	258 (51.1)	289 (53.9)	1407 (60.8)	1954 (58.2)
Screen time	382 (75.8)	349 (65.1)	1030 (44.5)	1761 (52.5)

*Values presented as n (%) unless specified differently.

†χ^2^ test for comparing differences in proportion of three age groups.

Regarding movement behaviour practices, children in the attending group had significantly higher PAT (21.4% vs 13.0%; *P* < 0.001), OT (88.7% vs 77.2%; *P* < 0.001), and SD (68.2% vs 59.8%; *P* < 0.001) during the last 24 hours, with similar patterns appearing during the last two weeks. However, ST among children in the attending group was significantly lower than in the non-attending group both during the last 24 hours (44.7% vs 51.2%; *P* = 0.001) and during the last two weeks (42.6% vs 52.9%; *P* < 0.001). For children aged 12-23 months, all movement behaviour indicators were significantly higher among children in the attending group than in the non-attending group, except for ST during the last 24 hours (*P* = 0.348). OT and SD in children aged 0-5 months during the last 24 hours and the last two weeks were significantly higher in the attending group, while children aged 6-11 months in the attending group showed higher OT and ST during the last 24 hours. Caregivers with children who attended ECE classes showed higher knowledge levels in OT, SD, and ST for all children (**Table 3**).

**Table 3:** Comparison of movement behaviours between children who attended (Attending group) and non-attended ECE classes (Non-attending group)*

	0-5 mo			6-11 mo			12-23 mo			Total		
	**Attending group (n = 302)**	**Non-attending group (n = 202)**	***P*-value**	**Attending group (n = 417)**	**Non-attending group (N = 119)**	***P*-value**	**Attending group (n = 1771)**	**Non-attending group (n = 544)**	***P*-value**	**Attending group (n = 2490)**	**Non-attending group (N = 865)**	***P*-value**
**Movement behaviour practices during the last 24 h**												
Physical activity time	37 (12.3)	18 (8.9)	0.302	100 (24.0)	24 (20.2)	0.455	396 (22.4)	70 (12.9)	<0.001	533 (21.4)	112 (13.0)	<0.001
Outdoor time	248 (82.1)	138 (68.3)	<0.001	385 (92.3)	100 (84.0)	0.011	1575 (88.9)	430 (79.0)	<0.001	2208 (88.7)	668 (77.2)	<0.001
Physical restraint	124 (41.1)	89 (44.1)	0.565	141 (33.8)	43 (36.1)	0.718	897 (50.6)	244 (44.9)	0.021	1162 (46.7)	376 (43.5)	0.113
Sleep duration	187 (61.9)	105 (52.0)	0.034	314 (75.3)	87 (73.1)	0.715	1197 (67.6)	325 (59.7)	<0.001	1698 (68.2)	517 (59.8)	<0.001
Screen time	208 (68.9)	144 (71.3)	0.632	238 (57.1)	81 (68.1)	0.041	668 (37.7)	218 (40.1)	0.348	1114 (44.7)	443 (51.2)	0.001
**Movement behaviour practices during the last two weeks**												
Physical activity time	53 (17.5)	25 (12.4)	0.148	109 (26.1)	23 (19.3)	0.161	418 (23.6)	98 (18.0)	0.007	580 (23.3)	146 (16.9)	<0.001
Outdoor time	267 (88.4)	151 (74.8)	<0.001	398 (95.4)	109 (91.6)	0.160	1612 (91.0)	458 (84.2)	<0.001	2277 (91.5)	718 (83.0)	<0.001
Physical restraint	108 (35.8)	89 (44.1)	0.075	109 (26.1)	36 (30.3)	0.439	722 (40.8)	183 (33.6)	0.003	939 (37.7)	308 (35.6)	0.288
Sleep duration	182 (60.3)	98 (48.5)	0.012	308 (73.9)	89 (74.8)	0.932	1177 (66.5)	325 (59.7)	0.005	1667 (66.9)	512 (59.2)	<0.001
Screen time	207 (68.5)	149 (73.8)	0.246	246 (59.0)	77 (64.7)	0.309	608 (34.3)	214 (39.3)	0.037	1061 (42.6)	440 (50.9)	<0.001
**Movement behaviour knowledge (caregivers know recommendations)**												
Physical activity time	25 (8.3)	14 (6.9)	0.700	66 (15.8)	21 (17.6)	0.738	299 (16.9)	78 (14.3)	0.180	390 (15.7)	113 (13.1)	0.074
Outdoor time	247 (81.8)	166 (82.2)	0.999	377 (90.4)	110 (92.4)	0.619	914 (51.6)	204 (37.5)	<0.001	1538 (61.8)	480 (55.5)	0.001
Physical restraint	199 (65.9)	130 (64.4)	0.795	261 (62.6)	84 (70.6)	0.134	1355 (76.5)	391 (71.9)	0.032	1815 (72.9)	605 (70.0)	0.105
Sleep duration	161 (53.3)	97 (48.0)	0.037	221 (53.0)	68 (57.1)	0.487	1116 (63.0)	291 (53.5)	<0.001	1498 (60.2)	456 (53.7)	<0.001
Screen time	221 (73.2)	161 (79.7)	0.117	269 (64.5)	80 (67.2)	0.660	784 (44.3)	246 (45.2)	0.733	1274 (51.2)	487 (56.3)	0.010

*Values presented as n (%) unless specified differently.

Almost all indicators of movement behaviour practices in the long NCT group were significantly higher than those in the short NCT group for both during the last 24 hours and during the last two weeks among all surveyed children. While ST in the long NCT group was higher than that in the short-NCT group for children aged 0-5 months during the last 24 hours (74.7% vs 61.1%; *P* = 0.002) and the last two weeks (76.2% vs 60.6%; *P* < 0.001); PR and ST were statistically significant indicators between the two groups in children aged 6-11 months (*P* < 0.05). In children aged 12-23 months, PAT was significantly higher in the long NCT group compared to the short NCT group during the last 24 hours (21.4% vs 18.0%; *P* = 0.045), while OT and ST also became significant alongside PAT during the last two weeks. Concerning movement behaviour knowledge in total children, caregivers who spend ≥4 hours/d on their child had a higher level of movement behaviour knowledge in all indicators (**Table 4**).

**Table 4:** Comparison of movement behaviour practices and knowledge between children with nurturing care time ≥4 h (long NCT group) and those with daily nurturing care time <4 h (short NCT group)*

	0-5 mo			6-11 mo			12-23 mo			Total		
	**Long NCT group (n = 324)**	**Short NCT group (n = 180)**	***P*-value**	**Long NCT group (n = 318)**	**Short NCT group (n = 218)**	***P*-value**	**Long NCT group (n = 1442)**	**Short NCT group (n = 873)**	***P*-value**	**Long NCT group (n = 2084)**	**Short NCT group (n = 1271)**	***P*-value**
**Movement behaviour practice for the last 24 h**												
Physical activity time	40 (12.4)	15 (8.3)	0.166	74 (23.3)	50 (23.0)	0.928	309 (21.4)	157 (18.0)	0.045	423 (20.3)	222 (17.5)	0.044
Outdoor time	255 (78.7)	131 (72.8)	0.132	290 (91.2)	195 (89.5)	0.498	1264 (87.7)	741 (84.9)	0.057	1809 (86.8)	1067 (84.0)	0.022
Physical restraint	147 (45.4)	66 (36.7)	0.058	123 (38.7)	61 (28.0)	0.010	719 (49.9)	422 (48.3)	0.478	989 (47.5)	549 (43.2)	0.016
Sleep duration	193 (59.6)	106 (58.9)	0.882	240 (75.5)	161 (73.9)	0.672	955 (66.2)	567 (65.0)	0.530	1388 (66.6)	834 (65.6)	0.558
Screen time	242 (74.7)	110 (61.1)	0.002	205 (64.5)	114 (52.3)	0.005	569 (39.5)	317 (36.3)	0.131	1016 (48.8)	541 (42.6)	<0.001
**Movement behaviour practice for the last two weeks**												
Physical activity time	47 (14.5)	31 (17.2)	0.419	73 (23.0)	59 (27.1)	0.278	354 (24.6)	162 (18.6)	<0.001	474 (22.7)	252 (19.8)	0.047
Outdoor time	274 (84.6)	144 (80.0)	0.192	305 (95.9)	202 (92.7)	0.102	1309 (90.8)	761 (87.2)	0.006	1888 (90.6)	1107 (87.1)	0.002
Physical restraint	133 (41.1)	64 (35.6)	0.226	99 (31.1)	46 (21.1)	0.010	589 (40.9)	316 (36.2)	0.026	821 (39.4)	426 (33.5)	<0.001
Sleep duration	183 (56.5)	106 (58.9)	0.601	244 (76.7)	153 (70.2)	0.089	957 (66.4)	545 (62.4)	0.054	1384 (66.4)	804 (63.3)	0.063
Screen time	247 (76.2)	109 (60.6)	<0.001	205 (64.5)	118 (54.1)	0.016	516 (35.8)	306 (35.1)	0.721	968 (46.5)	533 (42.0)	0.011
**Movement behaviour knowledge (caregivers know recommendations)**												
Physical activity time	26 (8.0)	13 (7.2)	0.747	58 (18.2)	29 (13.3)	0.128	254 (17.6)	123 (14.1)	0.026	338 (16.2)	165 (13.0)	0.011
Outdoor time	270 (83.3)	143 (79.4)	0.277	291 (91.5)	196 (89.9)	0.528	738 (51.2)	380 (43.5)	<0.001	1299 (62.3)	719 (56.6)	<0.001
Physical restraint	223 (68.8)	106 (58.9)	0.025	232 (73.0)	113 (51.8)	<0.001	1112 (77.1)	634 (72.6)	0.015	1567 (75.2)	853 (67.1)	<0.001
Sleep duration	173 (53.4)	90 (50.0)	0.465	195 (61.3)	94 (43.1)	<0.001	894 (62.0)	513 (58.8)	0.122	1262 (60.6)	697 (54.8)	0.001
Screen time	261 (80.6)	121 (67.2)	<0.001	221 (69.5)	128 (58.7)	0.010	650 (45.1)	380 (43.5)	0.468	1132 (54.3)	629 (49.5)	0.007

*Values presented as n (%) unless specified differently.

## DISCUSSION

This cross-section behavioural study examined the compliance with movement behaviour guidelines among children under two years old in urban China. We found that movement behaviour practices and knowledge were generally suboptimal. Moreover, children who attended ECE classes and whose primary caregivers’ nurturing care time was ≥4 hours/d had higher compliance with movement behaviour guidelines than their counterparts.

Our previous study in a rural area of Qinghai Province demonstrated that the status of healthy movement behaviours for children aged six to 20 months old was not promising [22]. Our new findings extend these results to a large sample of urban children in China. Compared with the study in Qinghai, we found a lower proportion of children meeting sedentary behaviour guidelines (consisting of physical restraint and screen time) in urban China, but higher proportions of children meeting outdoor time and sleep duration [22]. Besides regional differences, this inconsistency may result from the survey in Qinghai having been carried out during the COVID-19 pandemic, when the pandemic prevention and control measures limited outdoor activities [16]. Although this study was not nationally representative, it covered a wide range of geographic areas (31 provinces) and could provide data for understanding the current status of physical activity, ST, and sedentary behaviour of children under two years old in China. The proportions of children adhering to guidelines on physical activity (19.2%) and SD (66.0%) in our study were much lower than in Canada and Australia, where most children (99.3% and 82.1% in Canada and 96.5% and 79.7% in Australia, respectively) met the recommendations [18,30]. These large differences may be related to strategies for promoting parental knowledge and behaviours in different countries. For example, in contrast with Chinese caregivers, there may be more awareness of movement behaviour guidelines in Canada, which were released in collaboration with a communication organisation focused on promoting physical activity [30]. The low adherence to the movement guidelines among Chinese children is concerning, as compliance with these movement recommendations is associated with a reduced risk of obesity and other non-communicable diseases [5-8].

Recently, increasing numbers of Chinese children have attended classes in ECE institutions after birth [26]. We found that, compared to children who did not attend ECE classes, those who did had significantly higher compliance with PAT, OT, PR, and SD recommendations. ECE institutions are equipped with professional motor classrooms and equipment, such as balls or wheeled toys, which increase physical activity in children. Additionally, studies have shown that ECE teachers may set a positive example in healthy movement behaviours [31], and that playing with peers can also prolong physical activity time and promote outdoor activities [32]. However, we also found that the proportion of meeting ST recommendations in children who attended ECE classes is lower than in those who did not. Different from Western countries, parents in Asia believe that ST benefits their children’s learning [33]. Similarly, evidence has shown screen viewing for education was the greatest motivation for parents who reported not meeting guidelines [34]. Moreover, some parents may not be conscious of the potential health risks of screen viewing [35]. Consequently, we infer that primary caregivers of children who attended ECE classes have a strong desire for education, which results in more ST for their children to receive education. Hence, these findings suggested that ECE institutions have the potential for a significant public health impact to shape and improve movement behaviours in children. As young children are dependent on their caregivers for physical activity, the nurturing care time of primary caregivers is also crucial for improving adherence to guidelines, which we found to be higher among children whose primary caregivers’ nurturing care time ≥4 hours/d than their counterparts.

To the best of our knowledge, this study is the first large-scale cross-sectional survey to report compliance with movement behaviour guidelines for children under two years old in urban China. However, it has some limitations. First, we used self-reported questionnaires for data collection, possibly underestimating the true association with information bias in subjective measures [6]. Second, we used convenience rather than randomised sampling and only collected data through ECE institutes, so caution is advised when generalising the results [36].

## CONCLUSIONS

Our findings suggest that the status of movement behaviours for children under two years old in urban China is not optimistic, especially for PAT, PR, and ST, with over half of the children not meeting the recommendations. Attending early childhood education classes and primary caregivers’ daily nurturing care time is important for infants and young children to adhere to movement guidelines. Consequently, more ECE institution-specific practical strategies and educational materials are needed to promote compliance with movement behaviour guidelines, specifically restricting ST.
